# Multiparametric MRI identifies subtle adaptations for demarcation of disease transition in murine aortic valve stenosis

**DOI:** 10.1007/s00395-022-00936-5

**Published:** 2022-05-29

**Authors:** Christine Quast, Frank Kober, Katrin Becker, Elric Zweck, Jasmina Hoffe, Christoph Jacoby, Vera Flocke, Isabella Gyamfi-Poku, Fabian Keyser, Kerstin Piayda, Ralf Erkens, Sven Niepmann, Matti Adam, Stephan Baldus, Sebastian Zimmer, Georg Nickenig, Maria Grandoch, Florian Bönner, Malte Kelm, Ulrich Flögel

**Affiliations:** 1 Cardiovascular Research Laboratory, Division of Cardiology, Pulmonary Diseases and Vascular Medicine, Medical Faculty, Heinrich Heine University, Düsseldorf, Germany; 2 Aix‑Marseille Université, CNRS, CRMBM, Marseille, France; 3 Experimental Cardiovascular Imaging, Department of Molecular Cardiology, Heinrich Heine University Düsseldorf, Universitätsstraße 1, 40225 Düsseldorf, Germany; 4 Heart Centre Bonn, Clinic for Internal Medicine II, University Hospital Bonn, Bonn, Germany; 5 Clinic for Cardiology, University Hospital Cologne, Cologne, Germany; 6 Department of Pharmacology and Clinical Pharmacology, Heinrich Heine University, Düsseldorf, Germany; 7 CARID, Cardiovascular Research Institute Düsseldorf, Düsseldorf, Germany

**Keywords:** Aortic valve stenosis, MRI, Aortic flow patterns, Vessel wall remodelling, Tissue characterization

## Abstract

Aortic valve stenosis (AS) is the most frequent valve disease with relevant prognostic impact. Experimental model systems for AS are scarce and comprehensive imaging techniques to simultaneously quantify function and morphology in disease progression are lacking. Therefore, we refined an acute murine AS model to closely mimic human disease characteristics and developed a high-resolution magnetic resonance imaging (MRI) approach for simultaneous in-depth analysis of valvular, myocardial as well as aortic morphology/pathophysiology to identify early changes in tissue texture and critical transition points in the adaptive process to AS. AS was induced by wire injury of the aortic valve. Four weeks after surgery, cine loops, velocity, and relaxometry maps were acquired at 9.4 T to monitor structural/functional alterations in valve, aorta, and left ventricle (LV). In vivo MRI data were subsequently validated by histology and compared to echocardiography. AS mice exhibited impaired valve opening accompanied by significant valve thickening due to fibrotic remodelling. While control mice showed bell-shaped flow profiles, AS resulted not only in higher peak flow velocities, but also in fragmented turbulent flow patterns associated with enhanced circumferential strain and an increase in wall thickness of the aortic root. AS mice presented with a mild hypertrophy but unaffected global LV function. Cardiac MR relaxometry revealed reduced values for both T1 and T2 in AS reflecting subtle myocardial tissue remodelling with early alterations in mitochondrial function in response to the enhanced afterload. Concomitantly, incipient impairments of coronary flow reserve and myocardial tissue integrity get apparent accompanied by early troponin release. With this, we identified a premature transition point with still compensated cardiac function but beginning textural changes. This will allow interventional studies to explore early disease pathophysiology and novel therapeutic targets.

**Supplementary Information:**

The online version contains supplementary material available at 10.1007/s00395-022-00936-5.

## Introduction

Aortic valve stenosis (AS) is the most frequent valve disease in the elderly and accounts for left ventricular (LV) systolic and diastolic dysfunction as well as aortic wall distress. Thus, AS has a major socio-economic impact in the aging western society [52]. While therapeutic options are taken in severe AS by conventional or transcatheter valve replacement, the detailed underlying mechanisms of the disease and the interconnection between alterations of valve geometry and aortic wall shear stress are incompletely understood [41]. Furthermore, it is unclear whether monitoring of myocardial tissue texture changes or dynamic aortic flow patterns in AS might additionally drive therapeutic decisions [31, 47].

Lack of mechanistical insight is the main reason for missing early diagnostic and preventive measures. Although there is a variety of murine models available to study the development of calcific AS, a major obstacle to research in this area is that they require prolonged exposure (several weeks) to high fat/cholesterol diets and/or specific genotypes. Furthermore, most of them are characterized by calcific valvular sclerosis without hemodynamically significant stenosis [36, 50]. The latter is consistently developed only in transgenic mice expressing ApoB100 and concomitantly lacking the low‐density lipoprotein receptor [36]. To overcome these restrictions, investigations in a standardized system with experimentally induced AS are required. To this end, we made use of a recently described wire procedure [24] and refined this approach to a well-defined model of graded AS exerting major hallmarks of the disease, such as fibrosis, inflammation, and calcification of the valve, as confirmed by histology [40].

To utilize this model for exploration of pathophysiological mechanisms and identification of novel therapeutic targets, further longitudinal and highly reproducible imaging is required. In routine diagnostics, predominantly echocardiography serves as standard method for serial measurements to assess the degree and progress of stenosis, while CT is used for accurate planning of valve replacement [14]. MRI is able to add further reliable information concerning structure and function not only of the valve but simultaneously of the LV and the ascending aorta. Thus, in the present study we aimed to establish a multiparametric high-resolution MRI approach to reveal early, subtle changes in valvular, aortic, and ventricular morphology/function—encompassing assessment of myocardial, interstitial, and coronary compartments—before the manifestation of clinically overt disease characteristics. This will open the perspective to identify novel, premature imaging markers for disease onset, progression, and aggravation. In the translational setting, this will help to define new therapeutic targets and, ultimately, to more specifically guide decision making in treatment of AS.

## Methods

### Animals

For all experiments, male 12-week-old C57Bl/6 mice ranging from 20 to 28 g body weight (BW) were used. Animal experiments were performed in accordance with the Directive 2010/63/EU of the European Parliament on the protection of animals used for scientific purposes and the national guidelines on animal care. They were approved by the Landesamt für Natur, Umwelt, und Verbraucherschutz (LANUV, Nordrhein-Westfalen, Germany) under file reference 84-02.04.2017.A172. All animals used in this study were bred and kept at the central animal research facility of the Heinrich Heine University, Düsseldorf, Germany. They were fed with a standard chow diet and received tap water ad libitum. In total, *n* = 69 age-matched mice were analysed (33 animals with AS, 27 sham-operated animals (sham) and 9 age- and weight-matched untreated controls (con), 2 animals with severe aortic regurgitation showing a persisting mean diastolic backward flow ≥ 5 cm/s were excluded from analysis of AS.

### Surgical procedure

AS was induced as described previously [40]. In brief, mice were anesthetized by intraperitoneal injection with a mixture of ketamine (100 mg/kg BW) and xylazine (10 mg/kg BW). Thereafter, anaesthesia was maintained by 1.5–2.0% isoflurane. After endotracheal intubation, mice were mechanically ventilated and placed in a supine position on a warming pad. Thereafter, the right carotid artery was exposed, and a coronary wire (Universal; Abbott Cardiovascular, Plymouth, MN, USA) was inserted, advanced beyond the valve level and rotated as described before for induction of AS [40]. Afterwards, the right carotid artery was ligated. For sham operation, an identical surgery was performed, but the wire was not passed over the valve and rotations were performed above valve level. For post-surgical analgesia, 0.05–0.1 mg/ml buprenorphine in a maximum volume of 10 ml/kg body weight were applied subcutaneously every 6–8 h for 72 h following surgery.

### MRI

#### General

Data were recorded 28 days after wire injury at a Bruker AVANCE^III^ 9.4 T wide bore NMR spectrometer driven by ParaVision 5.1 (Bruker, Rheinstetten, Germany). Images were acquired using a Bruker microimaging unit Micro2.5 with actively shielded gradient sets (1.5 T/m) and a 25 mm quadrature resonator (Bruker) optimized for cardiovascular applications (Supplementary Fig. 1). Mice were anaesthetized with 1.5% isoflurane and kept at 37 °C. The front-paws and the left hind-paw were attached to ECG electrodes (Klear-Trace; CAS Medical Systems, Branford, CT, USA) and respiration was monitored by means of a pneumatic pillow positioned at the animal’s back. Vital functions were acquired by a M1025 system (SA Instruments, Stony Brook, NY, USA) and used to synchronize data acquisition with cardiac and respiratory motion. The entire scanning protocol took around 45 min and was well tolerated by all animals, which recovered within 2 min from anesthesia.

#### Cardiac cine MRI

For functional and morphometric analysis, high-resolution images of mouse hearts were acquired in short axis orientation using an ECG- and respiratory-gated segmented fast gradient echo cine sequence with steady-state precession (FISP). A flip angle (FA) of 15°, echo time (TE) of 1.23 ms, and a repetition time (TR) of about 6–8 ms (depending on the heart rate) were used to acquire 16 frames per heart cycle with an in plane resolution of 58.5 × 58.5 μm^2^; field-of-view (FOV), 30 × 30 mm^2^; matrix 512 × 512, slice thickness (ST), 1 mm; number of averages (NA), 3; zero-fill acceleration (ZFA), 2; acquisition time (TAcq) per slice for one cine loop, ~ 2.5 min. Routinely, 8–10 contiguous short axis slices were required for complete coverage of the LV. Screening for occurrence of necrosis was carried out by subsequent intraperitoneal gadolinium (Gd) contrast agent application (bolus of 0.2 mmol Gd-DTPA (diethylenetriaminepentacetate) per kg body weight). To this end, the animal handling system was shortly removed from the magnet and afterwards re-inserted with exactly the same positioning followed by scanning for regions with late Gd enhancement (LGE) as described previously [20]. For evaluation of functional parameters (e.g. end-diastolic and -systolic volume (EDV and ESV), ejection fraction (EF), etc.), ventricular demarcations in end-diastole and -systole were manually drawn with the ParaVision Region-of-Interest (ROI) tool. Beyond global cardiac function, we also analysed regional alterations to address as well local adaptive processes after induction of AS. To this end, we used an in-house developed software module based on LabVIEW (National Instruments, Austin, TX, USA), which divided the LV systematically into 200 equivalent sectors starting from the upper insertion point of the right ventricle as previously described (see Supplementary Fig. 2 for a more detailed description) [6, 20].

#### Aortic root

**Fig. 1:**
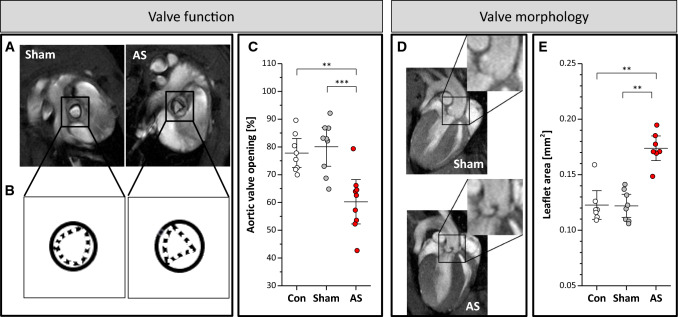
Valvular structure and function. **A + B** Valve opening in short axis views at the atrio-ventricular level in early systole in sham control (left) and AS (right) mice. **C** Opening of the aortic valve in untreated controls (con) sham-operated animals (sham), and mice subjected to wire injury expressed as percentage of maximal valve orifice in early systole to total inner aortic area in diastole. **D** Long axis views of AS (bottom) and sham (top) mice in early diastole revealing structural remodelling of the valve in AS. **E** Quantification of aortic leaflet area (see Supplementary Fig. 5); *n* = 9 each, ***P < 0.001

For analysis of the aortic valve, strain, and wall thickness, short axis cine loops were acquired at the atrio-ventricular level with the same parameters given above. Aortic valve opening was calculated from the maximal opening area in early systole related to the luminal area of the aorta in end-diastole (see Fig. 1 A+B). The cyclic deformation of the aorta upon cardiac ejection was utilized to determine the Green–Lagrange strain from the circumferential dimensions of the aorta in end-diastole (C_D_) and -systole (C_S_) by $E = \frac{1}{2}\left( {\frac{{C_{S}^{2} }}{{C_{D}^{2} }} - 1} \right)$ [38]; see also Supplementary Fig. 3 left.

These measures were further used to calculate the outer diastolic and systolic diameters of the vessel. Mean aortic wall thickness was estimated from the inner and outer borders of the vessel wall within the same cardiac time frame (Supplementary Fig. 3B). Here, both inner and outer areas were approximated by circles, and the difference between the two radii provided an averaged wall thickness of the aorta. Longitudinal slices orientated perpendicular to the atrio-ventricular level (see Supplementary Fig. 4 for localization) served to determine the total leaflet area as an estimate of aortic valve thickness (Supplementary Fig. 5). All demarcations were again manually drawn with the ParaVision ROI tool.

#### Velocity maps

Aortic flow profiles were obtained by acquisition of velocity maps at the atrio-ventricular level. Measurements were performed using an ECG- and respiration-triggered slice-selective fast low angle shot (FLASH) sequence with a four-point Hadamard scheme for flow velocity encoding [15]. Twelve frames per heart cycle were acquired using the following parameters: TE/TR, 1.75/7.50 ms; FA, 30°; FOV, 30 × 30 mm^2^; ST, 1 mm; matrix 256 × 256; NA, 4 resulting in a TAcq of ~ 5 min. For quantification of flow velocities, aortic demarcations were also manually drawn with the ParaVision ROI tool with output of mean and maximal velocities of the ROI. For 3D surface visualization, aortic flow profiles were extracted from the dataset by an in-house developed software module based on LabVIEW and plotted with OriginPro (Originlab Corporation, Wellesley Hills, MA, USA).

#### Relaxometric maps

Cardiac tissue characterization was carried out by T1 and T2 mapping essentially as described previously [6, 20]. T1 mapping was accomplished by variable flip angle analysis of retrospectively gated short axis slices (FA 2°, 5°, 8°, 11°, and 14°; TE of 1.26 ms; TR = 5.82 ms, FOV, 30 × 30 mm^2^; ST, 1 mm; matrix 256 × 256; NA, 100; ZFA, 2; TAcq of 1 min 16 s). Without the need of prospective triggering stable steady-state conditions are maintained as the repetition time remains unaffected by variations in cardiac and respiration rates [12, 20]. Acquisition of T2 maps was carried out using an ECG- and respiratory-gated multi-spin-echo sequence (16 echoes, separated by a ΔTE of 3.36 ms; TR = 500 ms, FOV, 30 × 30 mm^2^; ST, 1 mm; matrix 256 × 256; NA, 3; ZFA, 2; TAcq of ~ 6 min). Only one early ECG trigger per expiration phase was used to provide constant conditions for the recording of the echo train [6]. T1 and T2 maps were analysed by in-house developed software tools based on LabVIEW. For calculation of the extracellular volume (ECV, [19]), T1 maps were recorded before and after application of (Gd) contrast agent (see above). Immediately prior to MRI measurements, hematocrit was determined from 50 µl retro-orbitally collected EDTA blood using a VetScan HM5 (Abaxis Inc., Union City, USA).

#### Myocardial perfusion

For quantification of myocardial blood flow (MBF) we made use of cine arterial spin labeling (cine-ASL)—a technique that has been described in detail elsewhere [53, 54]. In brief, arterial spin labeling was applied to tag the longitudinal magnetization of blood water protons in the coronary arteries before they enter the imaging plane in the myocardium. For labeling, a slice-selective inversion slab was placed on the aortic root while another selective inversion slab was positioned in the opposite direction below the imaging slice for control labeling (Supplementary Fig. 6). Two ECG-gated midventricular short axis cine image series were acquired under labeled and control conditions with the following parameters: TE/TR, 1.33/6.13 ms; 15 frames per heart cycle, FA, 6°, FOV, 25 × 25 mm^2^; matrix, 128 × 64, ST, 1.5 mm, 25 cine blocks for both tag and control images (13 of the cine blocks were averaged), total TAcq ~ 6 min. Data were acquired under baseline conditions and after intraperitoneal bolus application of Regadenoson (0.2 mg per kg body weight [49]) for determination of the coronary perfusion reserve. Quantitative image analysis and calculation of perfusion maps were performed using in-house developed analysis tools built in the Interactive Data Language (IDL) as described previously [53, 54].

### Echocardiography

For validation of MRI-derived flow velocity data, a Fujifilm Visualsonics Vevo 3100 Ultra high frequency imaging platform (Toronto, ON, Canada) was utilized. Mice were anesthetized with 2% isoflurane under continuous monitoring of ECG, respiratory rate and body temperature. The chest of all mice was depilated and pre-warmed bubble-free ultrasound gel was applied to allow artefact-free image acquisition. Measurements were carried out in suprasternal view with a pulsed wave Doppler using angle correction between 45° and 55° as described in detail previously [40].

### Ex vivo analysis—histology, respirometry, autophagy, coronary flow reserve, and troponin

Animals were sacrificed after MRI by exsanguination in deep anaesthesia with xylazine (10 mg/kg BW) and ketamine (100 mg/kg BW), injected intraperitoneally. Blood collection for determination of circulating troponin levels was carried out via transthoracic heart puncture. Thereafter, hearts were excised and immediately washed with ice-cold buffer. Hearts were then prepared for Langendorff perfusion (see below) or transferred to a cryomold filled with embedding medium (Tissue-Tek; Sakura Fineteck, Staufen, Germany), frozen in liquid isopentane and stored at -80 °C for at least 1 week.

#### Analysis of the valve area and leaflet thickness

Hearts were cut at -20 °C into 6–8-µm slices using a Leica CM 3050 S cryostat (Leica, Wetzlar, Germany). For each heart, 8–10 representative slices of the aortic valve were collected on 5–6 adhesive slides (SuperFrost; Thermo Fisher, Waltham, USA). Before staining, the slides were air dried, dehydrated in a descending gradient alcohol series and finally washed in distilled water. After that, the slides were stained with haematoxylin and eosin (both Carl Roth, Karlsruhe, Germany) using standard procedures. After an ascending gradient alcohol series and 5 min clearing in xylene (Carl Roth), slices were embedded in Entellan (Sigma–Aldrich, St. Louis, Missouri, USA). After drying for at least 1 day, slides were analysed with a Leica DM4000M microscope, run with LasX software (Leica). Three representative pictures of each slice were taken at 10 × and 40 × magnification using a MC170HD camera. To quantify the morphological changes of the valve leaflets, the pictures were analysed using Fiji (Phi Gamma Delta Fraternity, Madison, USA). Measurement of the valve area was performed on one representative slice, in which the summed area of the three leaflets was assessed.

#### Immunofluorescence of valve and myocardium

Cryosections of 5 µm thickness were cut from embedded aortic valves or midventricular sections with the same cryostat as described above. Immunofluorescence staining was carried out after fixation with 4% paraformaldehyde (Carl Roth) in phosphate-buffered saline (PBS, Sigma) for 15 min and incubation in blocking solution (0.2% fish skin gelatine from cold water fish (Sigma), 0.5% BSA (Carl Roth), 0.1% saponin (Sigma) in PBS for 1 h at room temperature. Primary anti-vimentin antibody (Ab), ab45939 10 µg/ml; primary anti-α-smooth muscle actin (α-SMA) Ab, ab5694, 0.2 mg/ml; primary CD31 Ab, ab28364, 0.2 mg/ml (all Abcam, Cambridge, UK) were incubated in blocking solution at 4 °C overnight. Secondary Ab incubation was accomplished with goat anti-rabbit IgG (H + L) cross-absorbed secondary Ab, AlexaFluor488, A11008, or donkey anti-rabbit IgG (H + L) cross-absorbed secondary Ab, AlexaFluor594, A21207, 2 µg/ml (Invitrogen, Carlsbad, CF, USA). Staining with WGA Alexa Fluor™ 488 conjugate (Wheat germ agglutinin, Thermo Fisher Scientific Inc., Waltham, USA) was carried out after fixation with 4% paraformaldehyde (Carl Roth) in phosphate-buffered saline (PBS, Sigma) for 15 min. WGA was incubated in PBS 1 µg/ml at 4 °C for 10 min. For counterstaining of nuclei, VECTASHIELD Antifade mounting medium with DAPI (Vector Laboratories; Burlingham, USA) was used. Images were taken at 200 × magnification with an Olympus MX61 microscope and a 12-bit CCD monochrome (F-View II) camera driven by CellSense software (Olympus, Düsseldorf, Germany). For each animal, images of the three valve cusps and cusp bases were taken in one slice and two slices spaced by 30 µm were analysed per animal. Number of nuclei (stained with DAPI) and number of cells positive for vimentin or α-SMA were counted with Fiji. For determination of myocardial CD31 and cardiomyocyte diameter, three images were taken in two myocardial slices each, with slices spaced by 30 µm. To quantify myocardial CD31, the positive area was normalized to the number of nuclei in the region of interest. For determination of cardiomyocyte size, the diameter of 30 cardiomyocytes per image at height of nuclei (stained with DAPI) was assessed with Fiji.

#### Collagen and hyaluronan (HA)

Hearts were sliced in 5 μm sections followed by determination of collagen accumulation as detected by picrosirius red staining (0.1% picrosirius red in saturated picric acid solution), celestine blue solution (5% ammonium iron(III) sulfate, 0.25% celestine blue, 14% glycerine in DI water), and nuclei were stained as previously described [42]. HA detection was performed using a biotinylated bovine HA-binding protein (HAbP) (Calbiochem, Merck, Darmstadt, Germany, Cat. # 385911), and an HRP-conjugated streptavidin probe (Sigma–Aldrich St. Louis, MO, USA, Cat. # S5512).

#### Transmission electron microscopy

After excision and washing with ice-cold phosphate-buffered saline, four 2 mm^3^ blocks were cut from the ventricular septum in the basal part of the heart and immediately transferred to fixation buffer for thorough permeation of the tissue. After processing and cutting of the tissue following standard procedures, images were taken at a Hitachi H-7100 transmission electron microscope at the core facility for electron microscopy of the Heinrich Heine University Düsseldorf. Twenty images were taken at both 15.000 × and 30.000 × magnification. Number of mitochondria per field-of-view, area of mitochondria and percentage coverage of total image area by mitochondria as well as overall morphology of the tissue were assessed at 15.000 × magnification. Distance between single cristae and detailed morphology of mitochondria were analysed at 30.000 × magnification. Image analysis was performed with Fiji.

#### High-resolution respirometry

was carried out in saponine-permeabilized murine left ventricular apical myocardial fibers as previously described [45]. Mitochondrial respiration (oxygen flux) was quantified using an Oxygraph-2 k (Oroboros Instruments, Innsbruck, Austria) using substrate uncoupler inhibitor (SUIT) protocols and was normalized to tissue weight. Mitochondrial function was measured at 37 °C according to established protocols [46]. Oxygen flux was measured at saturating oxygen levels between 250 and 480 µM. The SUIT protocols included the following substances (in order of application). Protocol A (in mM): 2 malate, 10 glutamate, 2.5 ADP, 10 succinate, 0.01 cytochrome C, 0.005 oligomycin, stepwise titration of 0.2 µM carbonyl cyanide-trifluoromethoxyphenylhydrazone (FCCP), 0.4 µM rotenone, and 5 µM antimycin A. Protocol B (in mM): 2 malate, 1 octanoyl-carnitine, 2.5 ADP, 0.01 cytochrome C, 0.005 oligomycin, stepwise titration of 0.2 µM carbonyl cyanide-trifluoromethoxyphenylhydrazone (FCCP), 0.4 µM rotenone, and 5 µM antimycin A. The respiratory control ratio was assessed as oxidative phosphorylation capacity after addition of succinate (protocol A) or ADP (protocol B), respectively, divided by leak respiration after oligomycin. The integrity of the outer mitochondrial membrane was ensured by adding cytochrome C. An increase in oxygen flux by more than 15% after addition of cytochrome C led to exclusion of the experiment. Protocols were run in quadruplicates (protocol A) or duplicates (protocol B) and means were taken for the final results. As gold standard for quantification we normalised to the amount of mitochondria which have been evaluated by electron microscopy in the same animals applied to oxygraph analysis as described above.

#### Western blot analysis for autophagy and mitophagy

Cardiac tissue was lysed in radioimmunoprecipitation assay lysis buffer (RIPA). To each 10 ml of the RIPA Lysis Buffer one Pierce™ Protease and Phosphatase Inhibitor Mini Tablet (Thermo Scientific, Waltham, Massachusetts, USA) was added. Protein concentration was determined using the Bradford Reagent (Abcam®, Cambridge, UK) and bovine serum albumin (Carl Roth GmbH + Co. KG, Karlsruhe, Germany) as a standard with a FLUORstar Omega (BMG LABTECH GmbH, Ortenberg, Baden-Württemberg, Germany). After the protein concentration was measured, equal amounts of protein (50 µg) were loaded on a Q-PAGE precast Bis–Tris 4–12% Gel (SMOBIO, Hsinchu City, Taiwan). Proteins were separated by electrophoresis (70 V for 30 min and 120 V for up to 2 h) and transferred onto an Amersham™ Protran™ Nitrocellulose Blotting membrane (Cytiva life sciences, Freiburg im Breisgau, Germany). The blots were then stained with the Revert™700 Total Protein Stain (LI-COR, Lincoln, Nebraska, USA) and imaged with the LI-COR Odyssey Fc (LI-COR, Lincoln, Nebraska, USA) to confirm the protein loading. After destaining the membranes with Revert™700 Destaining (LI-COR, Lincoln, Nebraska, USA), they were blocked for 1 h with Intercept™ Blocking Buffer (LI-COR, Lincoln, Nebraska, USA). Subsequently, the membranes were incubated with OPA1-L Rabbit mAb (1:1000, #80471S, Cell Signaling, Boston, USA), ATP5A1 Polyclonal Antibody (1:1000, #PA5-27,504, Invitrogen, Waltham, Massachusetts, USA), COX IV Rabbit Ab (1:1000, #4844S, Cell Signaling, Boston, USA) or Rabbit anti-LC3B pAb (1:1000, #NB100-2220, NOVUSBIO, Littleton, Colorado, USA) shaking overnight at 4 °C. The membranes where then washed and incubated for 1 h at room temperature with the secondary antibody IRDye 800CW Goat anti-Rabbit IgG (1:20,000, #926–32,211, LI-COR, Lincoln, Nebraska, USA). The bands were visualized using the LI-COR Odyssey Fc (LI-COR, Lincoln, Nebraska, USA) and protein expression levels were determined and quantified by measuring the band intensity and normalized to the total protein staining using the Empiria Studio™ Software (LI-COR, Lincoln, Nebraska, USA). The final results were presented as ratios. For the quantification of the ratio of LC3B II to LC3B I (LC3B-II/I) ImageJ was used as an analysis tool (https://imagej.net/software/fiji/).

#### Langendorff perfusion

For ex vivo analysis of the coronary flow, hearts were attached to a Langendorff apparatus (Hugo Sachs Electronics, March-Hugstetten, Germany) by cannulation of the ascending aorta. Thereafter, perfusion of the isolated hearts with modified Krebs–Henseleit buffer was immediately started. A balloon was introduced for left ventricular pressure measurement and an electrode was fixed for pacing the hearts at a constant frequency of 600 min^−1^. After an equilibration period of 20 min, heart perfusion was stopped for 1 min and then restarted to quantify the reactive coronary flow increase after global ischemia. The maximal coronary flow value after re-initiation of perfusion was then compared to the baseline flow before global ischemia and expressed as percentage increase.

#### Troponin

Withdrawn blood was collected in a heparinized 1 ml syringe (B. Braun Melsungen AG, Melsungen, Germany). Within 15 min thereafter, the blood was centrifuged at 10.000*g* for 15 min at room temperature. Plasma was collected and frozen at − 80 °C until analysis. High-sensitivity troponin T assays (Roche Diagnostics, Basel, Switzerland) were performed by the Central Institute for Clinical Chemistry and Laboratory Diagnostics, University Hospital Düsseldorf, according to the instructions of the manufacturer.

### Statistics

Only mice without relevant aortic regurgitation were included into statistical analysis, which was carried out by OriginPro. All data are given as mean values ± standard deviation (SD). Data were analysed for Gaussian distribution using Kolmogorov–Smirnov normality test. A one-way ANOVA with Tukey's multiple-comparisons test or a two-tailed t test were used to determine significant differences between groups. *P* values ≤ 0.05 have been considered as statistically significant.

## Results

### Aortic valve morphology and degree of valvular impairment

High-resolution MRI was carried out 4 weeks after induction of AS. Using a segmented FISP sequence with prospective gating, cine loops at the atrio-ventricular level were acquired for assessment of valve morphology with accurate assignment to early and late diastole and systole, respectively, allowing an exact planimetry of the valve in all phases of the cardiac cycle (Fig. 1, Supplementary Movie 1). Sham-operated (sham) and age-matched untreated mice (con) showed a homogenous and unrestricted opening of the valve resulting in an opening area of 80.1 ± 8.6% and 77.8 ± 6.4%, respectively, as normalized to the total inner supravalvular aortic area in end-diastole (beginning of the QRS complex). As expected, in mice subjected to wire injury we observed a more triangular orifice and an impaired valve opening of only 60.3 ± 9.8% (*P* < 0.001, *n* = 9 each group), which is comparable to human morphology in moderate degenerative AS (Fig. 1A–C, Supplementary Movie 1). Besides detection of the restricted orifice, high-resolution long axis slices acquired perpendicular to the aortic valve also revealed an altered shape and thickening of the valve as consequence of the remodelling processes induced by the surgical procedure (0.18 ± 0.02 vs. 0.12 ± 0.02 mm^2^, *P* < 0.001, *n* = 9 each group; Fig. 1D + E; Supplementary Movie 2). Subsequent histology confirmed the in vivo findings of valve thickening and revealed that this was accompanied by incipient fibrosis as indicated by enhanced vimentin and α-smooth muscle actin staining (Supplementary Fig. 7).

**Fig. 2:**
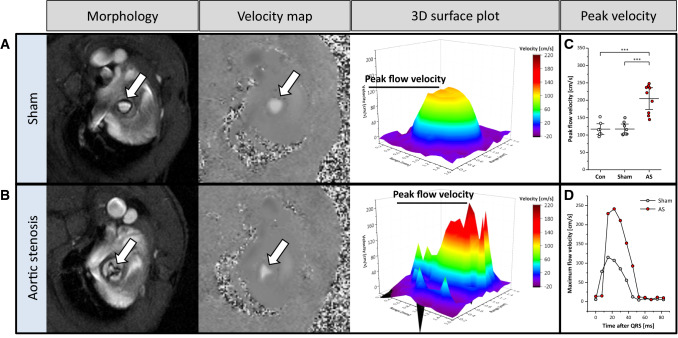
Aortic flow profiles. **A + B** Anatomical reference (left) and 2D velocity maps (middle) for assessment of blood flow at the level of the opened valve in sham controls (top) and AS (bottom). Velocity flow profiles were measured over the vessel cross section and are displayed as 3D surface plots in early systole (right). Peak velocity was higher in AS (**C**) and occurred later after valve opening as shown for two representative time courses of AS and sham-operated animals (**D**). Arrows indicate valve orifice; *n* = 9 each, ***P < 0.001

The extent of stenosis is usually estimated by determination of peak flow velocity across the valve by echocardiography. Here, we used MRI velocity maps to reconstruct cine flow profiles above the valve over the entire cardiac cycle (Fig. 2, Supplementary Movie 3). While sham-operated mice showed a bell-shaped flow profile in early systole, AS resulted not only in significantly enhanced peak flow velocities (204.8 ± 38.9 vs. 111.9 ± 26.4 cm/s; *P* < 0.001, n = 9 each group; Fig. 2C), but also in fragmented turbulent patterns of the flow profile which included isolated spikes of strongly accelerated velocities and as well areas with negative flow peaks (Fig. 2A + B). Note, that due to the increased resistance for blood output through the reduced orifice, peak velocity is achieved later in mice with AS compared to sham controls as shown in representative time courses in Fig. 2D. Parallel echocardiographic examinations corroborated the magnitude of flow increase in AS animals determined by MRI (~ 100 cm/s, Supplementary Fig. 8), while absolute values acquired by Doppler echocardiography tended to be higher in all groups—a phenomenon already reported earlier in comparison to 2D phase-contrast measurements [44]. Of note, dispersion of data and SDs were quite similar for MRI and echocardiography in all groups.

### Revealing aortic regurgitation via black jets and flow pattern

**Fig. 3:**
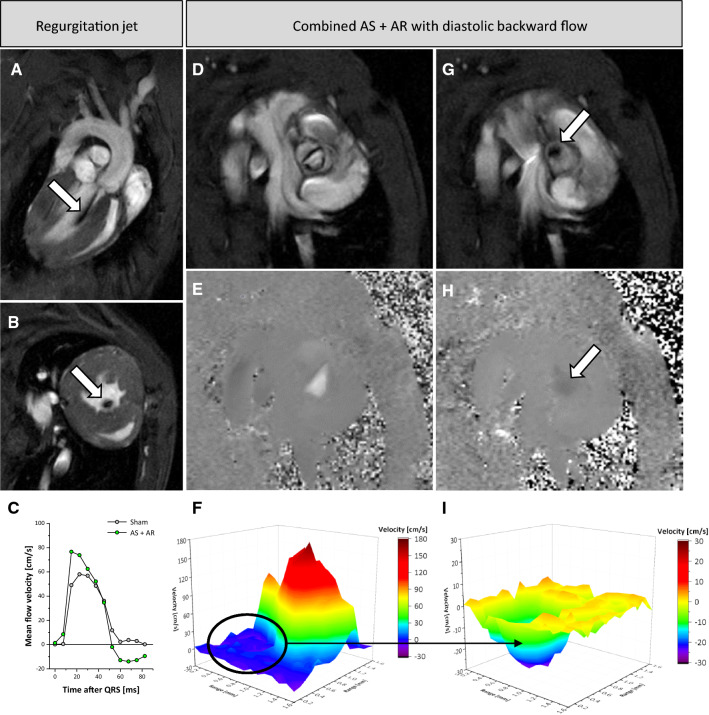
Aortic regurgitation (AR) combined with AS. In cine loops, backward flow due to regurgitation is reflected by black jets immediately after valve closure at the beginning of relaxation (arrows in **A + B + G**). Long axis view (**A**) and short axis views at the mid ventricular (**B**) and valvular level (**G**)—the latter compared to the same view in early systole (**D**). **E + H** Corresponding velocity maps to (**D + G**) show a slight dark backflow at the location of impaired valve opening (arrow in **H**). Quantification revealed negative backflow in combined AS + AR and an early rise due to pendular volume (**C**). 3D visualization of asymmetric flow patterns over the stenotic valve in early systole (**F**) and diastolic backflow after valve flapping (**I**). For the sake of clarity, in (**H**) the contrast was doubled as compared to the velocity maps displayed in (**E**) and the colour coding in (**I**) adapted to the negative flow

Aortic regurgitation (AR) might occur as consequence of the remodelling process after induction of AS or due to the surgical intervention itself. In MRI cine loops, the emerging backward flow is reflected by black jet artefacts in early diastole immediately after valve closure (Fig. 3A + B + G, Supplementary Movie 4). However, this is a rather qualitative measure and the magnitude of this artefact strongly depends on the imaging plane and its orientation to the backward flow. Thus, we used the created cine flow profiles for a more quantitative evaluation of the degree of AR (Fig. 3C + E + F + H + I). With this, diastolic backward flow could be precisely determined and mice with AS only easily discriminated against the combined case with AR. Depending on the individual remodelling process after induction of AS the resulting valve morphology led to asymmetric opening and regurgitation patterns. In an interesting example, functionally bicuspid AS (Fig. 3D–F) was associated with AR exactly at the same position where valve opening was restricted (Fig. 3G-I). Here, we also observed an accelerated increase in mean flow velocity caused by enhanced slew rate due to pendular volume (Fig. 3C). Nevertheless, the majority of stenotic valves in this model (> 90%) exhibited no or only a mild component of AR. Only two animals showed a persisting mean diastolic backward flow ≥ 5 cm/s and were excluded from analysis of AS due to severe AR.

### Aortic wall strain and morphology

**Fig. 4:**
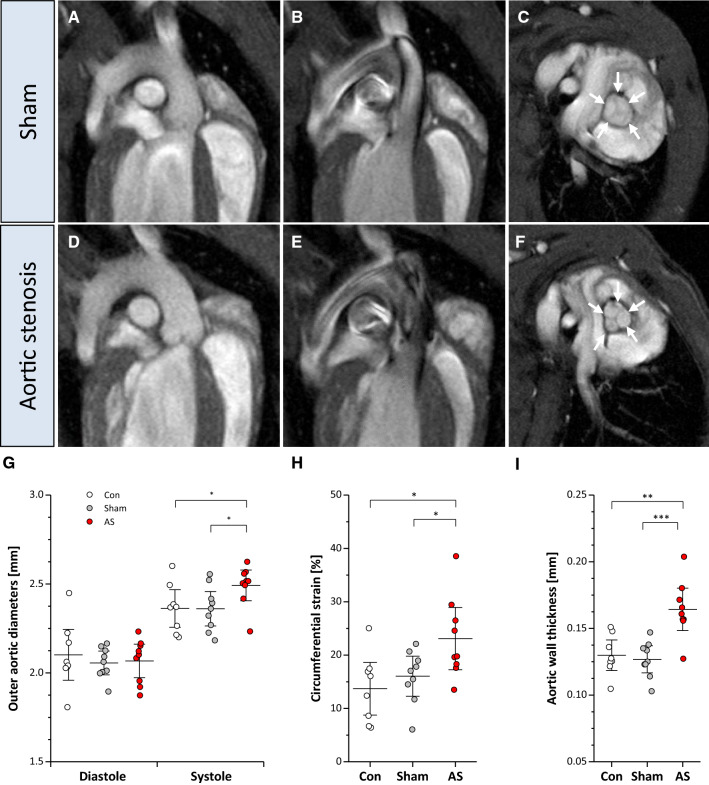
Aortic wall strain and morphology. **A + D** Aortic arch in end-diastole in sham controls and AS. **B** While sham mice exhibited a homogenous flow pattern in the ascending aorta in early systole, in AS turbulent flow occurred (black patchy flow artefacts) (**E**), which resulted in increased systolic distension (**G**) and enhanced circumferential strain in AS (**H**). **F + I** As a consequence, mice with AS displayed a thickening of the aortic wall (arrows) at the valvular level as compared to controls (**C**); *n* = 9 each, *P < 0.05, **P < 0.01

While an unrestricted valve opening led to a homogenous blood flow coaxial to the vessel wall (Fig. 4A + B; Supplementary Movie 2), aortic valve stenosis resulted in turbulent flow above the valve as reflected by irregular black jet artefacts in the ascending aorta (Fig. 4D + E; Supplementary Movie 2). The altered ejection patterns were associated with an increased distension of the vessel as reflected by significantly enhanced systolic diameters and circumferential strain in the aortic root of AS mice (2.49 ± 0.11 vs. 2.36 ± 0.12 mm and 23.1 ± 7.2 vs. 16.0 ± 4.6%, respectively; *P* < 0.05, *n* = 9 each group; Fig. 4G + H). As a consequence of the raised long-term burden within the outflow tract, 4 weeks after surgery aortic wall thickness was significantly increased in AS mice as compared to sham-operated controls (0.16 ± 0.02 mm vs. 0.12 ± 0.02 mm; *P* < 0.001, *n* = 9 each group; Fig. 4C + F + I).

### LV functional and structural changes

**Fig. 5:**
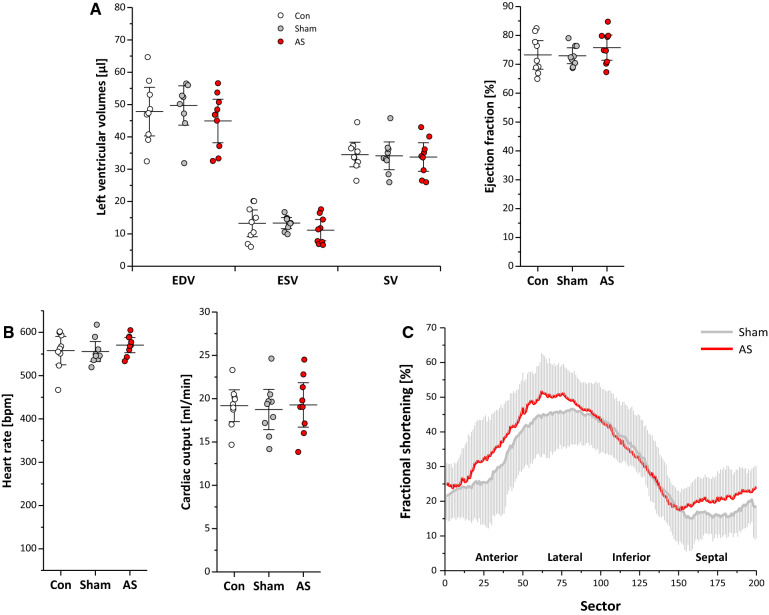
Left ventricular function. **A + B** Global left ventricular volumes, ejection fraction and cardiac output were unaltered in AS mice. **C** Regional analysis of cardiac function (see Supplementary Fig. 2) showed a trend to an increased fractional shortening in AS except for the inferior wall but without reaching the level of significance. For the sake of clarity, SDs are plotted in light grey and data of untreated controls were omitted; *n* = 9 each

**Fig. 6:**
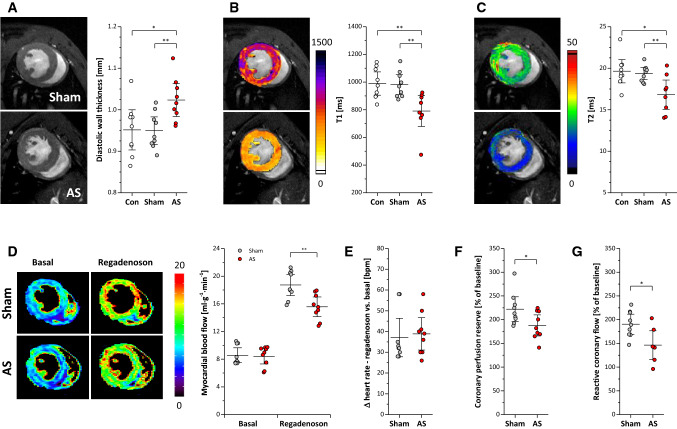
Myocardial morphology, tissue texture and perfusion. **A** Anatomical reference images demonstrated a significantly increased diastolic wall thickness in AS mice, which was accompanied by reduced myocardial T1 and T2 values as revealed by MR relaxometry (**B + C**). **D–F** cine-ASL revealed restricted perfusion reserve in AS mice upon Regadenoson application despite similar effects on heart rate in both groups; data in (**D**) are mean MBFs in diastole, n = 9 each, *P < 0.05, **P < 0.01. **G** Quantification of coronary flow reserve in Langendorff-perfused hearts upon reactive hyperemia for *n* = 6–9 animals per group; *P < 0.05

To assess the impact of AS in the present model on the LV, short axis cine loops were acquired for analysis of morphological and functional parameters (Fig. 5+6A Supplementary Movie 5). Of note, global cardiac function in terms of EF and cardiac output was unaffected 4 weeks after surgery (Fig. 5A + B). End-diastolic and -systolic volumes remained unchanged excluding mice presenting with severe AR (data not shown). The regional analysis of the LV (see Supplementary Fig. 2) indicated an increased fractional shortening in all sectors except for the inferior wall but without reaching the level of significance (Fig. 5C). However, for diastolic wall thickness, we observed a mild but significant increase in AS compared to sham-operated controls (1.02 ± 0.05 vs. 0.95 ± 0.04 mm; *P* < 0.05, *n* = 9 each group) indicating a mild hypertrophy of the LV wall (Fig. 6A). In line with this, LV mass showed a trend to increase but without reaching significance (data not shown).

Cardiac tissue characterization by T1 and T2 mapping surprisingly revealed reduced values for both parameters in AS as compared to sham controls (T1: 791 ± 136 vs. 981 ± 88 ms; *P* < 0.01, *n* = 9 each group; T2: 16.8 ± 2.1 vs. 19.4 ± 0.9 ms; *P* < 0.01, *n* = 9 each group; Fig. 6B + C). Concomitantly, we found no signs for myocardial LGE after application of Gd-based contrast agents (data not shown). Additional acquisition of post-contrast T1 maps also provided no evidence for any alterations in cardiac extracellular volume of AS mice (ECV 23.3 ± 6.9 vs. 25.3 ± 4.9% in sham controls, *n* = 9 each group). This clearly argues against development of significant myocardial oedema or necrosis at this time point but indicates subtle structural alterations in the hearts of AS mice owing to the increased afterload. In a final step, cine arterial spin labeling (cine-ASL [54]) was used to assess in the present model the impact of AS on myocardial perfusion. As can be recognized in Fig. 6D, baseline myocardial blood flow was similar in both groups, but upon Regadenoson stimulation we found a small, but significantly lower perfusion increase in AS mice (Fig. 6D, Supplementary Movie 6) despite the drug induced in both groups a comparable increase in heart rates (~ 40 bpm, Fig. 6E). Of note, a similar restriction in coronary perfusion reserve as observed in vivo (Fig. 6F) was also detected ex vivo upon reactive hyperemia in Langendorff-perfused hearts of AS and sham mice (Fig. 6G).

**Fig. 7:**
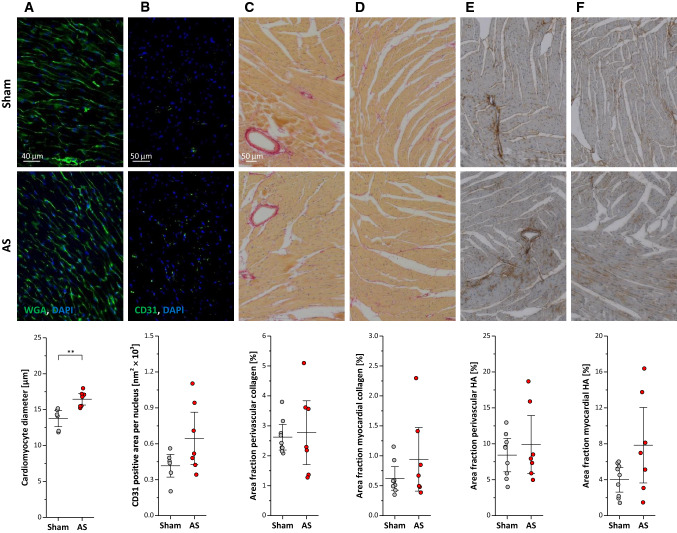
Myocardial histology. Top and middle: Histologic sections of midventricular myocardium stained for (**A**) wheat germ agglutinin (WGA), (**B**) CD31 as marker for capillarization, (**C + D**) collagen, and (**E + F**) hyaluronan as major component of the extracellular matrix (ECM). Bottom: Quantification reveals significant increased cardiomyocytes and a trend towards increased myocardial capillarization in AS compared to sham mice with no signs of interstitial or perivascular fibrosis and/or ECM remodelling; *n* = 5–9 each, **P < 0.01

Subsequent histology demonstrated that the moderate thickening of the LV wall in AS observed in ^1^H MRI cine loops was related to an increased cardiomyocyte size (∅ 16.4 ± 1.1 vs. 13.7 ± 1.4 µm; *P* < 0.01, Fig. 7A). This was associated by a trend towards higher capillarization in AS mice (Fig. 7B, *P* = 0.08), but without indications of interstitial or perivascular fibrosis and/or remodelling of the extracellular matrix (ECM), respectively (Fig. 7C–F, *n*  = 5–9 each group). In parallel, we found the subtle morphological alterations described above to be accompanied by approximately doubled troponin levels in the blood of AS mice as compared to sham controls (1.15 ± 0.55 vs. 0.48 ± 0.17 mg/ml; *P* < 0.05).

**Fig. 8:**
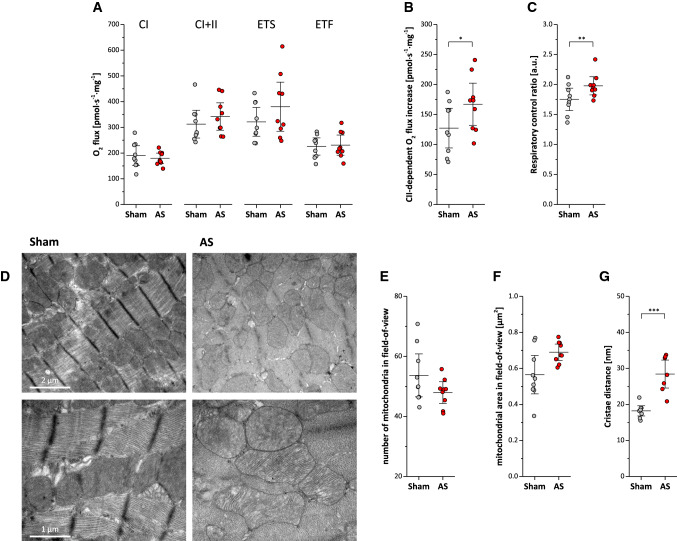
High-resolution respirometry and electron microscopy. Top: Respirometry of left ventricular myocardial fibers showing (**A**) O_2_ flux through complex I (CI), complex I + II (CI + II), electron transport system (ETS), electron transfer flavoprotein (ETF), (**B**) Succinate/CII-dependent O_2_ flux increase and (**C**) respiratory control ration of AS and sham mice; n = 10–11 each, *P < 0.05, **P < 0.01; all data were collected using Protocol A (see Method section), except for the ETF (far right in **A**) for which Protocol B was utilized. Bottom: Electron microscopy indicates beginning disorganization of cardiomyocyte ultrastructure in AS mice (**D**) with a trend towards less and larger mitochondria (**E** + **F**) and an increased distance between cristae (**G**); *n* = 9 each, ***P < 0.01

**Fig. 9:**
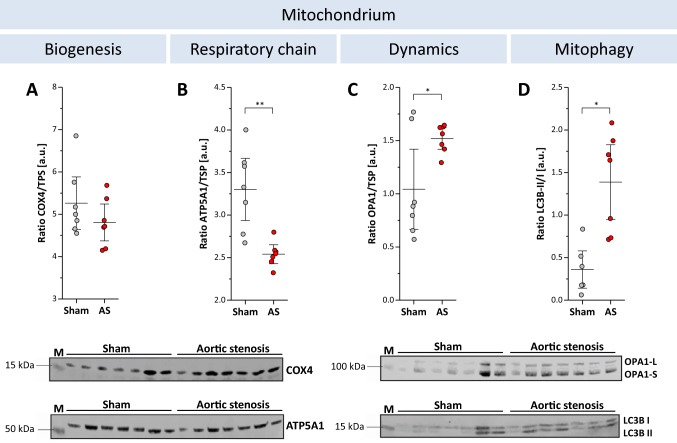
Alterations in mitochondrial proteins. Top: Quantification of western blots from proteins involved in (**A**) mitochondrial biogenesis (COX4), (**B**) respiratory chain (ATP5A1), (**C**) energy dynamics (OPA1) and (**D**) mitophagy (ratio LC3B-II/I), *n* = 7 each group, *P < 0.05; **P < 0.01.). Bottom: Representative original blots. *TPS* total protein staining; *a.u.* absolute units; *M* marker; *kDA* kilodalton

Furthermore, we observed that myocardial oxidative capacity and electron transfer system capacity did not differ between animals with and without AS as determined by high-resolution respirometry (Fig. 8A). However, succinate-dependent oxygen flux increase, when added after malate, glutamate, and ADP, was higher in AS than in sham (162.8 ± 45.2 vs. 123.2 ± 45.9 pmol⋅s^−1^⋅mg^−1^; *P* < 0.05; Fig. 8B). In addition, AS animals exhibited a higher respiratory control ratio (a marker of mitochondrial coupling) as compared to shams (1.99 ± 0.19 vs. 1.72 ± 0.23 a.u.; *P* < 0.01; Fig. 8C; n = 10–11 each group) suggesting further intracellular adaptations to the altered hemodynamic state. Electron microscopy indicated that this was associated with a beginning disorganization of the cardiomyocyte ultrastructure (Fig. 8D). While total mitochondrial area was unchanged compared to sham controls (43.8 ± 4.7 vs. 42.8 ± 5.9% of the field-of-view, n = 9 each group), we noted a tendency to less but larger mitochondria (Fig. 8E + F, *P* = 0.12 + 0.11) with incipient distributional imbalance of mitochondria and a significantly increased distance between cristae (28.4 ± 5.0 vs. 18.2 ± 1.8 nm; *P* < 0.001). Finally, western blot analysis was employed to monitor alterations in proteins involved in mitochondrial biogenesis (COX4), respiratory chain (ATP5A1), energetics (OPA1), and mitophagy (ratio LC3B-II/I; Fig. 9A–D, *n* = 7 each group). While no differences were detected for COX4, ATP5A1 was significantly decreased in AS vs. sham (2.54 ± 0.15 vs. 3.30 ± 0.48 a.u.; *P* < 0.01). On the other hand, both LC3B-II/I ratio (sham vs. AS: 0.36 ± 0.29 vs. 1.39 ± 0.57 a.u.; *P* < 0.01) and OPA1 (sham vs. AS: 1.04 ± 0.49 vs. 1.52 ± 0.13 a.u.; *P* < 0.05) were significantly increased in AS.

## Discussion

In the present study, we elaborated a murine AS model that exerts major hallmarks of the human disease, such as fibrosis, inflammation, and calcification of the valve as characterized by histology. Within this model, we applied high-resolution MRI at 9.4 T to simultaneously evaluate incipient changes in myocardial, valvular, perfusion, and aortic pathophysiology in a multiparametric fashion. Already 4 weeks after induction of AS, we could unveil subtle alterations in all three compartments which cannot be provided by any other non-invasive technology. With this approach, we were able to characterize a premature transition period with a variety of compensatory mechanisms maintaining cardiac function but also with incipient impairments of the myocardium including mitochondrial function and coronary flow reserve. This will open the perspective (i) for early interventional and mechanistic studies in genetic mice models to identify new targets in early disease progression and to gain more insight into the developing pathophysiology of AS, (ii) to detect adverse myocardial disease processes at early stage, and (iii) to develop novel imaging markers particularly for the still challenging topic of appropriate therapy and assessment of disease progression in apparently asymptomatic aortic valve stenosis.

### Valve morphology and function

High-resolution MRI enabled reliable analysis of valve opening in controls and in mice with AS by accurate planimetric delineation of the aortic valve orifice over the entire cardiac cycle. The provided image quality of valvular structures and orifice area facilitated correct plane recording of all valve cusps with high reproducibility despite diminutive proportions of the valve. Consistent with human valvular disease [48], we identified with this approach functionally bicuspid stenotic aortic valves (e.g. Figure 3). In this case, accompanying AR resembled a coaptation defect due to an immobile rather than a ruptured cusp, which cannot be clearly distinguished by echocardiography due to limits in spatial resolution. Besides assessment of valve morphology, we were also able to delineate valve thickening as a consequence of tissue remodelling, which was confirmed by subsequent histology. However, we have clearly to admit that evaluation of valve thickness is somewhat restricted by pixel size. Compared to histology, leaflet area determined by MRI was overestimated by approximately 30% (~ 0.12 vs 0.09 mm^2^ in AS) which is most likely caused by (i) imprecise orthogonal orientation of the longitudinal MR imaging plane to the valve and (ii) averaging its area over a slice thickness of 1 mm (and not only 6–8 µm as in histologic sections) and thereby along the valve curvature over the leaflets to the cusp. Despite these limitations, the magnitude of valve thickening (~ 0.1 mm^2^) found by MRI and histology was quite similar in AS mice, which offers the option for non-invasive longitudinal and serial monitoring of subtle changes in valve morphology of murine disease models.

### Impact on aortic flow profiles

As expected, mice with AS were characterized by an increase in maximum peak velocity comparable to echocardiographic assessment. In line with previous observations in humans [44], absolute values assessed by 2D phase-contrast measurements tended to be lower in all groups, but the extent of flow increase in AS animals (~ 100 cm/s) almost matched the Doppler findings. Furthermore, we identified fragmented turbulent flow patterns which were shifted to substantially higher peak flow velocities as consequence of valve orifice narrowing. The present approach could further be extended to segmented analysis of flow patterns in the entire aorta, which permits full visualization of valves and their inflow/outflow tracts. In contrast, quantification of transvalvular flow by echocardiography is challenging because velocities must be measured in line with the transducer beam, making it susceptible to errors caused by misalignment of the transducer beam to the direction of the blood flow. This is highly relevant in eccentric and dynamic flow jets and essential for reliable quantification of AS [5].

Accurate flow quantification furthermore allows a precise measure of the degree of regurgitation as well as cardiac shunt volumes/ratios and differential flow volumes [39]. In our model, it was possible to identify regurgitation flow as black jets in cine loops and to quantify the resulting backflow across the valve in corresponding velocity maps. With these techniques, we were able to easily differentiate between mice presenting with AS only and AS combined with AR. By future implementation of 4D-flow techniques, also eccentric regurgitation jets could be assessed by multidirectional acquisition encoding all components of the velocity vector [8, 32, 51].

### Morphology and function of the supravalvular ascending aorta

As a consequence of the altered flow patterns in AS, we observed enhanced circumferential strain in mice with AS which was associated with an increase in aortic wall thickness at the level of the aortic root. This implies relevant structural and functional aortic changes in our AS model. Aortic strain in tricuspid aortic stenosis has been assumed to be a diagnostic measure of aortic stiffness in patients [57], but in contrast to findings in humans with bicuspid aortic valve [34], we observed an enhanced circumferential strain in the ascending aorta in animals with functionally bicuspid aortic valve (Fig. 4H). This is most likely due to the pronounced turbulent flow pattern with strongly enhanced radial velocity components and a still intact windkessel function in this early phase of AS.

Of note, the same considerations mentioned above for measurement of valve thickness apply for determination of the dimensions of the aortic wall. Again, the restrictions caused by pixel size and partial volume effects lead to a systematic overassessment as compared to histology [56]. Nevertheless, our results were quite robust for the individual groups investigated and the magnitude of the observed increase in wall thickness (~ 30%) is in the same order as in histologic examinations of a hypertensive mouse model with pathological remodelling of the aorta [28]. Furthermore, also circumferential strains derived from those measures were in pretty good agreement with a previous reference study on morphometry and strain distribution of the C57BL/6 mouse aorta by ultrasound [18].

### Myocardial adjustments to the increased afterload

While we observed a gradual wall thickening of the LV myocardium, LV function was interestingly still preserved 4 weeks after induction of AS without any signs of necrosis (no LGE) or alterations in the ECV. By trend fractional shortening was enhanced in all LV wall areas of AS mice (except for the inferior wall), revealing an early compensatory mechanism together with the mild LV hypertrophy. At the same time, we observed reduced T1 and T2 relaxation times in our AS model, which preclude the presence of myocardial fibrosis and oedema at this stage, but rather reflect early adaptation processes. Histology confirmed the absence of any interstitial/perivascular fibrosis and/or remodelling of the ECM in AS mice and revealed an increased cardiomyocyte size as underlying cause of the observed wall thickening. Concomitantly, while all groups displayed similar maximum oxidative capacity, we observed both an enhanced complex II-dependent respiration and respiratory control ratio in mitochondria of AS mice. This indicates an increased relative ATP yield via ATP synthase for the myocardium [11, 25] and, thus, most likely represents another intracellular compensatory mechanism in response to the extended energy demand for pumping against the elevated afterload [1]. On the other hand, raised activation of complex-II-associated respiration may be accompanied by enhanced release of reactive oxygen species [21] which could be causative for the observed incipient distributional and microarchitecture disorders in the mitochondrial apparatus. Of note, these were also accompanied by adaptions at the protein level impacting on respiratory chain (down-regulation of ATP5A1), energetic efficiency (up-regulation of OPA1) and mitophagic processes (increased ratio of LC3B-II/I) [7, 17, 29]. Importantly, these alterations in tissue texture can provide a simple explanation for the observed decrease in both relaxation times: The beginning disorganization of the usually tightly packed cardiomyocyte ultrastructure leads to increased local tissue inhomogeneity, which is well known to cause faster proton dephasing and affects T1 and T2 in the same way [27, 59]. Thus, it is tempting to speculate that the uncommon simultaneous drop of cardiac T1 and T2 might be useful as a premature in vivo readout for mitochondrial disorders in the heart, which could be even more sensitive than conventional parameters of myocardial damage, such as ECV, LGE, etc. [33].

Together, the induced myocardial adjustments seem to be sufficient to keep LV function at this moment in a compensated state reflecting the latent asymptomatic period of AS also known from human pathophysiology [9]. Nevertheless, despite a trend to an enhanced myocardial capillarization in AS mice, we observed in vivo and ex vivo a significant restriction in coronary flow reserve well known to be associated with AS in humans [23, 35, 60] and as well a mild increase in circulating troponin levels. Obviously, the current time point represents a period of transition, where a variety of compensation mechanisms still keeps cardiac function in balance but the persisting increased afterload already leads to incipient impairment of coronary flow and cardiomyocyte ultrastructure.

Further investigations are required to define more precisely the point in time when the present model turns into the onset of severe symptoms, such as depressed LV function and its sequelae [26]. Thereafter, progressive fibrosis and oedema are expected to reverse the somewhat surprising results of reduced T1 and T2 in our AS mice [4, 5]. However, the early observation of these subtle alterations clearly highlights the potential of the present MR approach to identify early changes in cardiac tissue texture and premature transition points in the adaptive process to AS thereby fostering future mechanistic studies in genetic mice models.

### Methodological advance

**Fig. 10:**
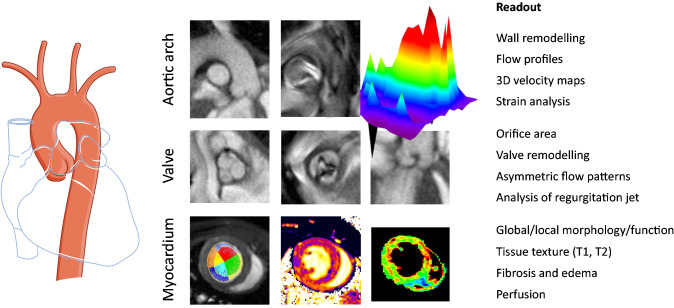
Summary of the benefits of MRI for a holistic analysis of AS covering heart, valve, ascending aorta and aortic arch as a mechanistical unit. Left: Anatomical scheme; middle: Representative sample MRI data at the level of aortic arch, valve and myocardium from sham-operated and AS mice; right: Tabular listing of MRI readouts within the target structure

There are rare reports about MRI with focus on aortic valve disease in mice [37, 43, 55], but the comprehensive and quantitative characterization of transvalvular aortic flow profiles together with structural/functional/perfusion changes in aortic valve, LV, and the ascending aorta over the entire cardiac cycle for a holistic analysis of the impact of AS in mice is unique and has not been carried out so far. With this, it is feasible to treat myocardium, valve, ascending aorta and aortic arch together as a mechanistical unit (Fig. 10) and to study pathophysiological changes in the context of complex aortovalvular diseases, such as AS, in all compartments in a serial and simultaneous manner. While echocardiography is established to determine the manifest grade of AS via assessment of aortic flow velocity, our MRI approach allows not only to delineate alterations in flow patterns and valve anatomy but also subtle and early adaptations in tissue texture (T1/T2 mapping), perfusion patterns, and functional feedback loops in parallel (see above). The reliable characterization of structural components such as the annulus, left ventricular outflow tract, aorto-coronary coupling, and valvular morphological changes itself are key features of our imaging approach and highly relevant for future optimization of diagnostic and therapeutic options in aortovalvular diseases.

### Beyond (murine) AS models

There are a number of animal models of calcific aortic valve disease (CAVD) recapitulating distinct human hallmarks in diet-induced, genetic, congenital, and developmental mouse as well as rabbit and porcine models, each with its specific restrictions [22, 50]. While diet-induced models often resemble changes in metabolic disorders with mild valvular impairment, some mice and rabbit models acquire hemodynamically significant calcific aortic valve disease, but the same has yet to be shown in pig for long-term follow up [2]. Moreover, it has to be noted, that some hemodynamic situations from mice are not transmissible to human parameters and need to be considered during analysis, e.g. high resting heart rate of about 600 bpm and, therefore, reduced heart rate reserve. On the other hand, while a translational approach with large animal models is closer to human dimension, it entails other restrictions, e.g. only few options for genetic manipulation. In each case, the animal needs to be immobilized during the acquisition phase and general anesthetic regimens have the potential to affect cardiac function [3]. However, to avoid cardiodepressant side effects [30], in our case isoflurane levels were kept at the lowest possible concentration for a stable sleep and a valid assessment of cardiac function and perfusion.

### Future perspectives and translational outlook

Concerning the reliability of echocardiography and MRI to determine the severity of aortic valve stenosis, many comparisons have been conducted and the results from these clinical studies support the assumption, that both techniques are in good agreement [13, 51, 58]. Beyond altered morphology and flow patterns, the major pathophysiological features of AS comprise inflammation, fibrosis, and calcification. While evaluation of the latter is clearly the domain of CT and ^18^F-NaF-PET, MRI offers also the possibility to detect more subtle events which precede the overt myocardial and valvular phenotype of AS, that are inflammatory and fibrotic processes. Additional application of Gd-based contrast agents (CAs) would allow detection of both replacement and interstitial fibrosis by late gadolinium enhancement and determination of extracellular volumes via T1 mapping, respectively. Furthermore, chemical exchange saturation transfer techniques have recently been applied to monitor remodelling of the extracellular matrix, while ^19^F MRI in combination with fluorinated CAs permits the background-free visualization of infiltrating immune cells [16, 42]. This bridges the gap to analysis of pathophysiologic pathways and elucidation of potential therapeutic targets in transgenic mouse models. In the clinical setting, this may be helpful to delineate and predict (bioprosthetic) valve degeneration which is expected to become a progressively important issue in the near future [10]. To this end, MRI may be combined with complementary methods (micro-CT, PET-CT, etc.) for a premature identification of disease initiation via altered flow profiles and micro-calcification allowing an early therapy of incipient degenerative processes.

Given that the described MRI approach has the potential to elucidate more subtle changes of the valve itself, the aortic wall and the affected myocardium, this can be expected to provide valid markers for the early diagnosis of incipient adverse myocardial and aortic remodelling allowing a timely initiation of the required therapy. Furthermore, combination of a reproducible animal model of AS with high-end longitudinal imaging will facilitate a better mechanistical understanding of AS which ultimately could lead to identification of critical transition points in disease development at the valvular, myocardial, and aortic level, and in turn to generation of novel pharmacological treatment options in humans. Thus, the further refinement of MRI techniques in well-defined preclinical longitudinal AS studies is a critical prerequisite for the translation of future imaging approaches to guide treatment of disease in affected patients.

## Conclusions

In summary, we demonstrate that in experimental AS high-resolution MRI provides important information on structural and functional aspects of valve, aorta, myocardium, and coronary perfusion. This comprehensive imaging approach with distinct reproducibility offers repetitive and highly sensitive assessment of subtle adaptions to disease and provides the basis for future analysis of (i) incipient pathophysiological mechanisms underlying the progression of AS itself and (ii) concomitant hemodynamic, aortic, and myocardial adaption processes with the perspective to evaluate novel therapeutic interventions targeting premature transition points in early disease development.

## Supplementary Information

Below is the link to the electronic supplementary material.

Supplementary file1 (PDF 777 KB)

Supplementary file2 Supplementary Movie 1: Short axis cine loops at the atrio-ventricular level. (MP4 3521 KB)

Supplementary file3 Supplementary Movie 2: Long axis cine loops. (MP4 6670 KB)

Supplementary file4 Supplementary Movie 3: Compilation of cine loops, velocity maps and 3D surface plots. (MP4 6091 KB)

Supplementary file5 Supplementary Movie 4: Detection of AS with aortic regurgitation. (MP4 5716 KB)

Supplementary file6 Supplementary Movie 5: Midventricular short axis cine loops. (MP4 1505 KB)

Supplementary file7 Supplementary Movie 6: Midventricular cine-ASL loops. (MP4 3085 KB)

## Data Availability

All data supporting the findings of this study are available within the article and its supplementary material.
