# Supplementary material for: Multiparametric MRI identifies subtle adaptations for demarcation of disease transition in murine aortic valve stenosis
Source: Basic Res Cardiol. 2022 May 29;117(1):29. doi: 10.1007/s00395-022-00936-5 (PMC9148878; doi:10.1007/s00395-022-00936-5)
Supplement: Supplementary file 1 — Supplementary file1 (PDF 777 KB) [file 395_2022_936_MOESM1_ESM.pdf]

Supplementary Figure 1

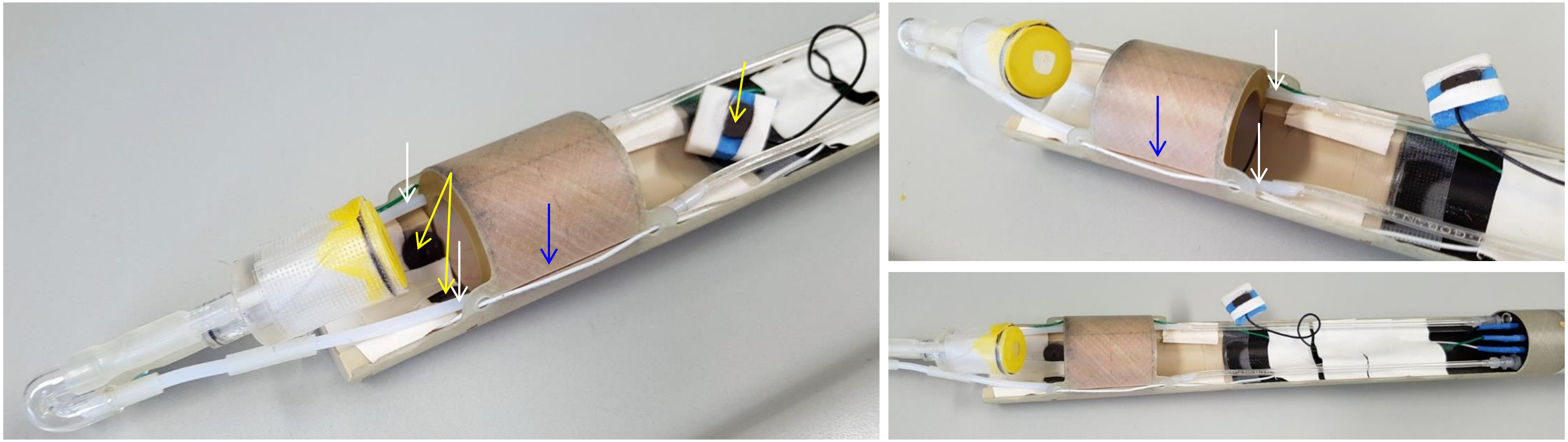

Design of the quad-resonator for cardiovascular applications

Since vertical wide bore MR spectrometers are usually installed without Faraday cage, the use of ECG electrodes inside the magnet is prone to cause antenna problems resulting in zipper artefacts and substantially reduced SNR in the MR images. This can be overcome by placing the leads of the electrodes around the resonator in tiny grooves at the outside of the probe (blue arrows). The subsequent fixing of the ECG electrodes for the front/hind-paws just before/behind the resonator (yellow arrows) avoids any radiofrequency interferences. Passing the inlet/outlet tubes for the anaesthesia gas through the wall of the resonator in between its coils (white arrows) both improves the field homogeneity and saves some space for the object of interest within the sensitive volume.

Images show different views of the resonator (25/40 mm inner/outer diameter) with anaesthesia mask (with inlet and outlet) as well as front- and hind-paw electrodes (yellow arrows) connected to the ECG adapters of the probe (right bottom: blue pins).

Supplementary Figure 2

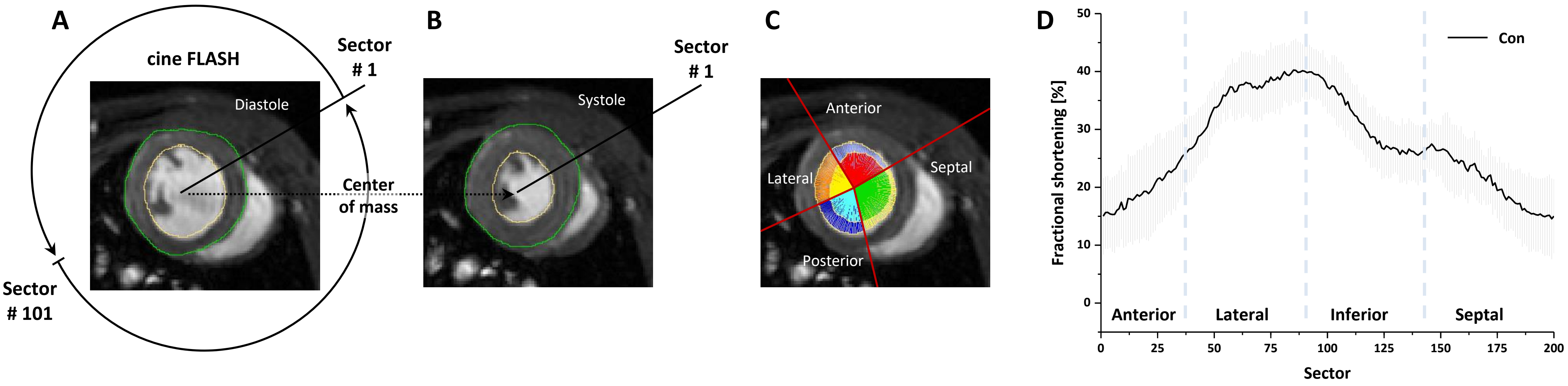

Regional wall movement analysis over 200 sectors

Local fractional shortening (FS) was calculated from the cine loops acquired in short-axis slices orientation. End-diastolic **(A)** and end-systolic **(B)** borders of the cavity were delineated and inner radii starting from the end-diastolic centre of mass were calculated before and after contraction. The left ventricle was then divided into 200 equally distributed sectors starting from the anterior insertion of the right ventricle (sector #1). Thereafter, FS was automatically calculated in each sector by an in-house developed software as the difference of end-diastolic and end-systolic radii divided by the end-diastolic value and displayed in radial charts **(C)**. Note, that sectoral wall contraction can easily be assessed visually from the distance between diastolic and systolic endocardial radii in the radial FS chart. As expected, FS strongly depends on wall region. **(D)** Averaging over the indicated sectors demonstrated largely different values for FS over the different wall areas, with decreasing FS from lateral, posterior, anterior to septal sectors. Data are means ± SD from n=9 control animals. For sake of clarity, SDs are shown within the full sector plots as shadows. Adapted from references 12 + 13.

Supplementary Figure 3

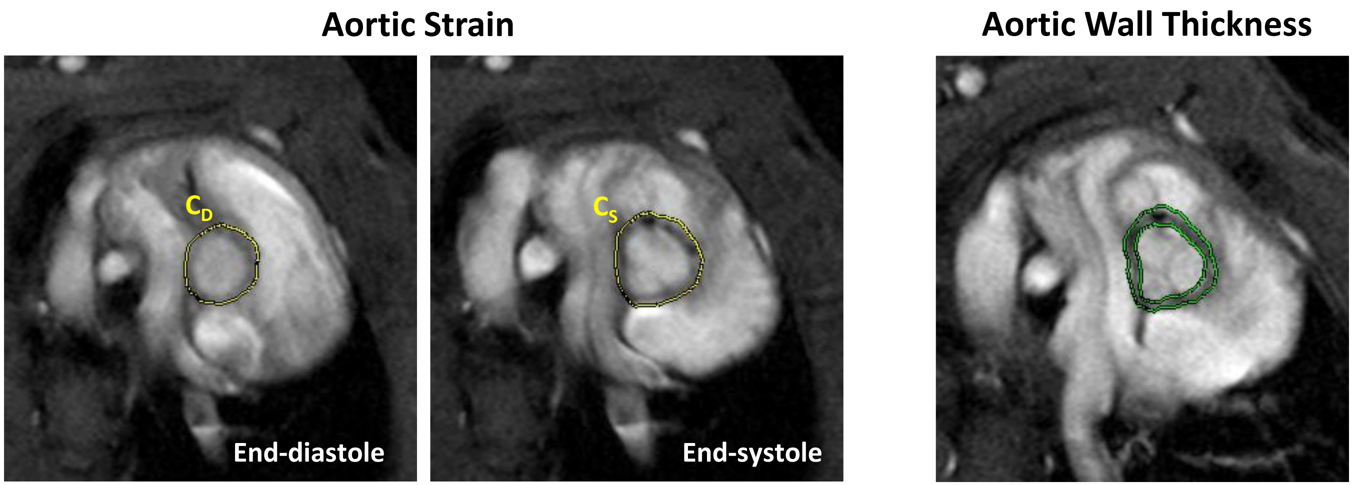

MRI quantification of aortic strain, diameters and wall thickness

Left: The cyclic deformation of the aorta upon cardiac ejection was utilized to determine the Green-Lagrange strain from the circumferential dimensions of the aorta in end-diastole (C_D) and end-systole (C_S).[12] These circumferences were further used to calculate the diastolic and systolic outer diameter of the vessel. **Right:** Mean aortic wall thickness was estimated from the inner and outer borders of the vessel wall. For calculation, both inner and outer areas were approximated by circles, and the difference between the two radii provided an averaged wall thickness of the aorta. All demarcations were manually drawn with the ParaVision Region-of-Interest (ROI) tool.

Supplementary Figure 4

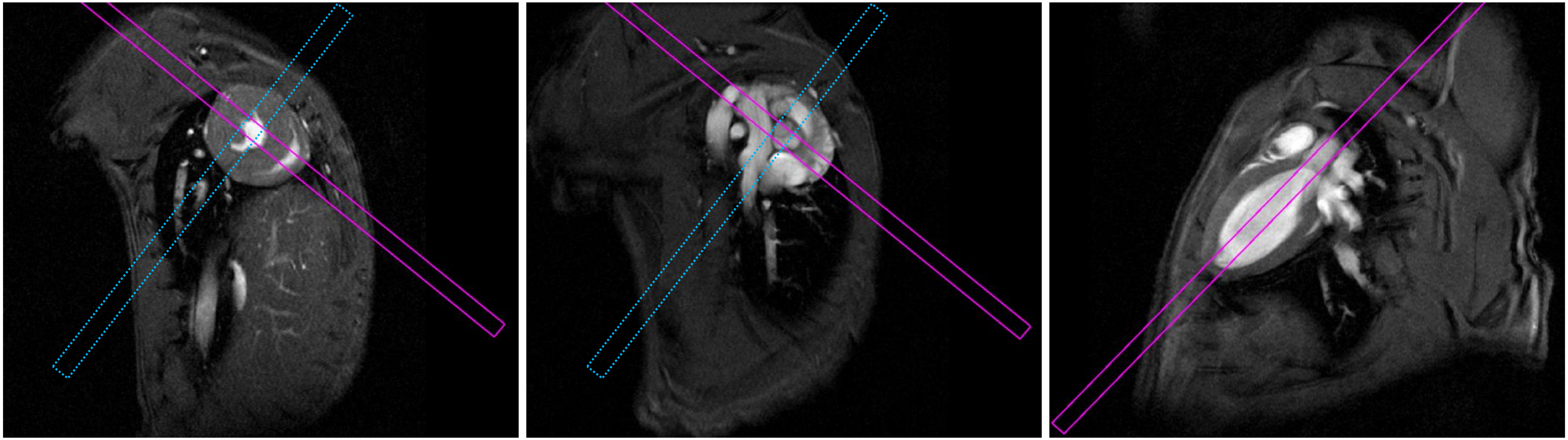

Orientation of Cardiac Long Axis Slices

Longitudinal slices were orientated perpendicular to midventricular short axis slices (left) and the atrio-ventricular level (middle) as indicated by the solid rectangles. Adequate coverage of the aortic outflow tract was additionally confirmed in orthogonal view (right; orientation shown by doted rectangles in left and middle images).

Supplementary Figure 5

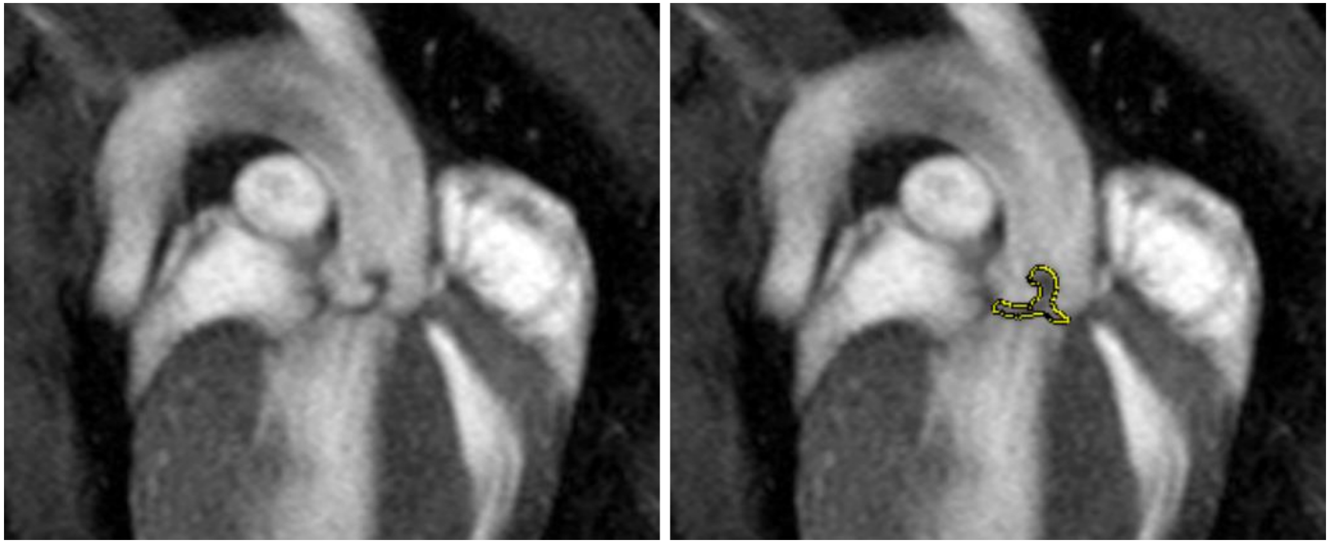

MRI quantification of aortic leaflet area

Longitudinal slices orientated perpendicular to the atrio-ventricular level (see Additional Figure 4 for localization) served to determine the total leaflet area as an estimate of aortic valve thickness. Demarcations (right) were manually drawn with the ParaVision Region-of-Interest (ROI) tool.

Supplementary Figure 6

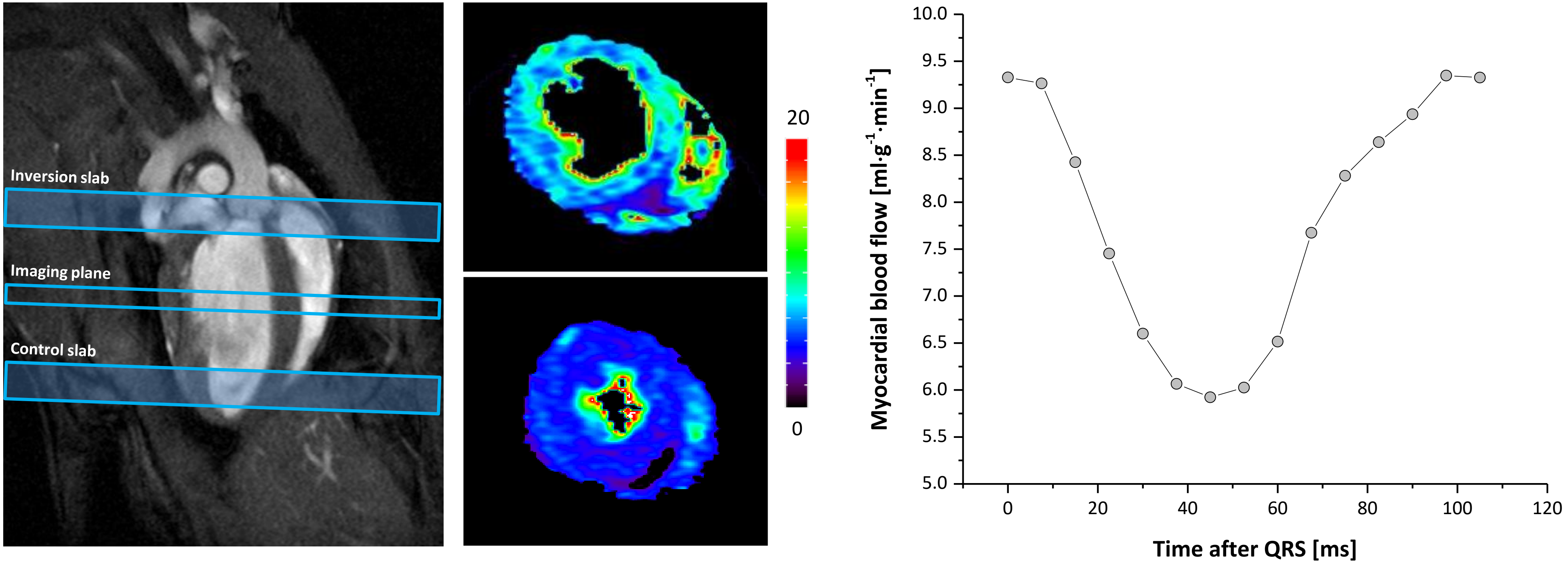

Cine arterial spin labeling for quantification of myocardial blood flow (MBF)

Left: Arterial spin labelling was applied to tag the longitudinal magnetization of coronary blood water protons before it enters the imaging plane in the myocardium. For labelling, a slice-selective inversion slab was placed on the aortic root while another selective inversion slab was placed in the opposite direction below the imaging slice (as control labelling). Middle: Representative color-coded MBF images in end-diastole (top) and –systole (bottom). Right: Typical MBF time course recorded for sham animals under baseline conditions. Data provided in Figure 6 represent mean MBFs in diastole.

Supplementary Figure 7

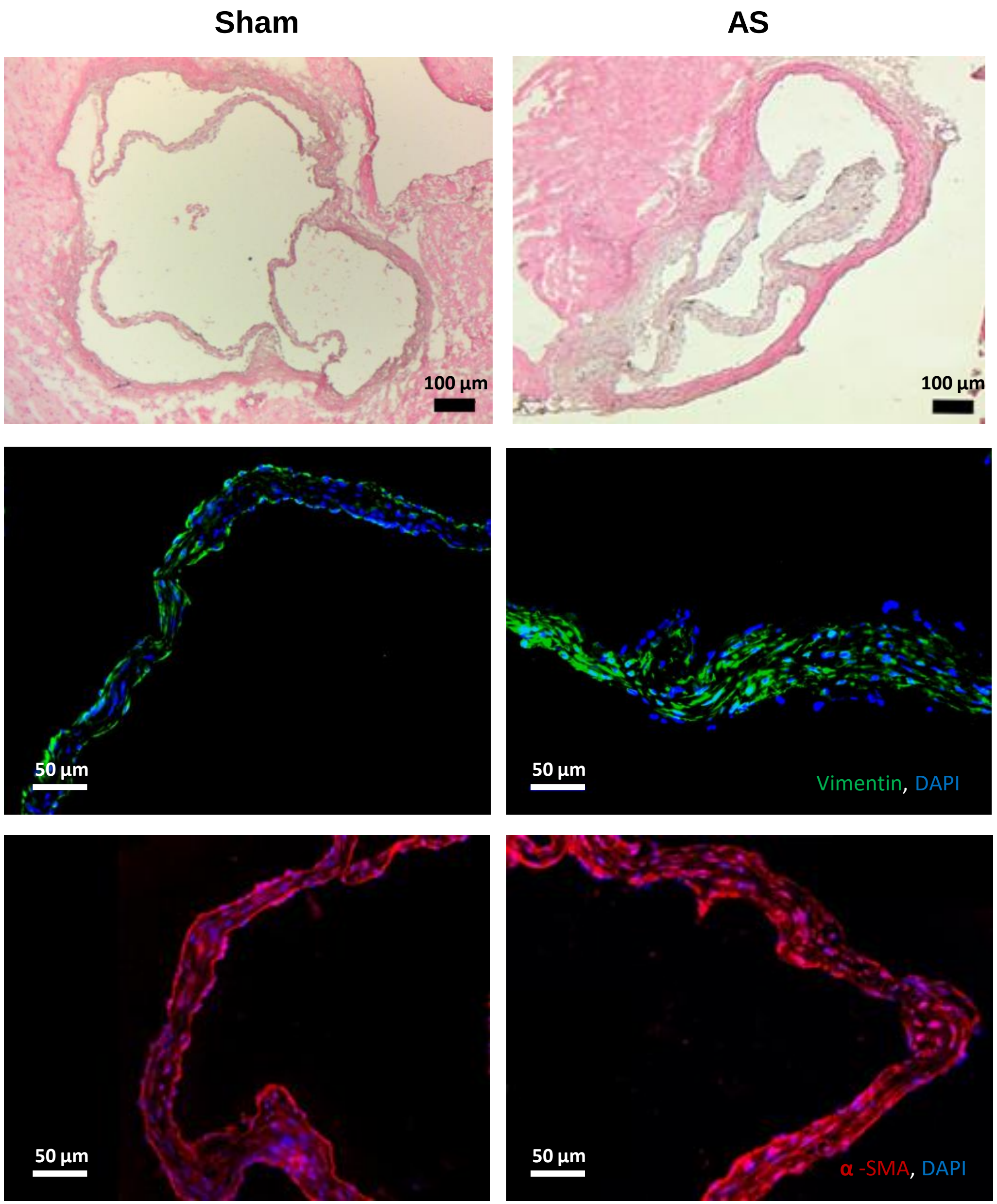

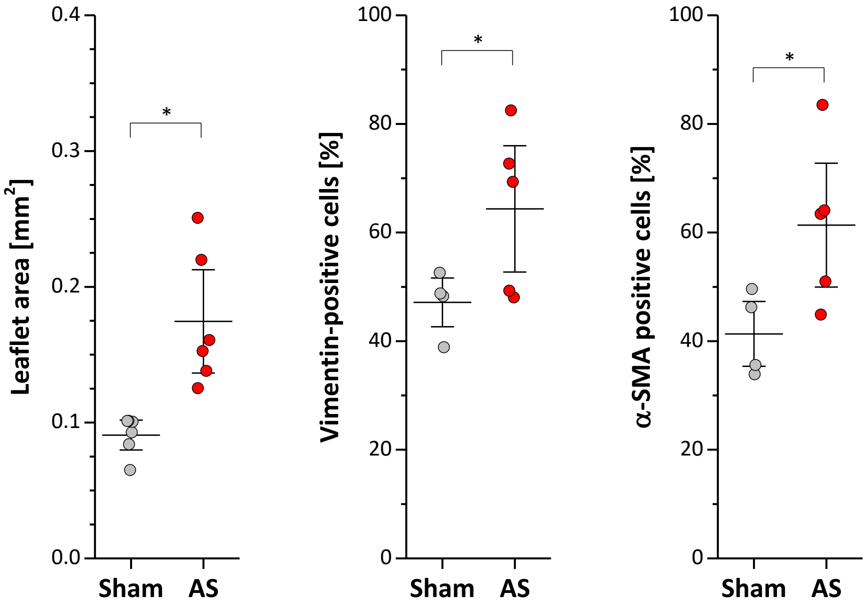

Validation of aortic stenosis by histology

Left: Histologic sections of aortic valves stained with hematoxylin and eosin (H&E, top) as well as for vimentin (middle) and α-smooth muscle actin (α-SMA, bottom). Images demonstrate fibrotic remodelling in AS animals with enhanced valve thickness and increased amount of cells positive for vimentin (green) and α-SMA. **Right:** Quantification for n=4-6 animals per group; **P*<0.05.

Supplementary Figure 8

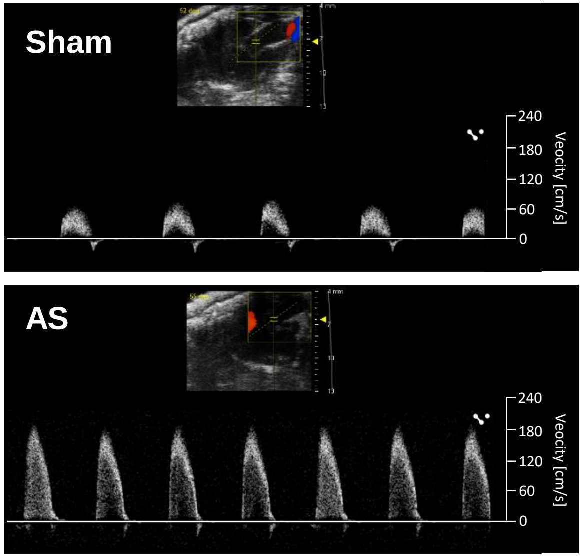

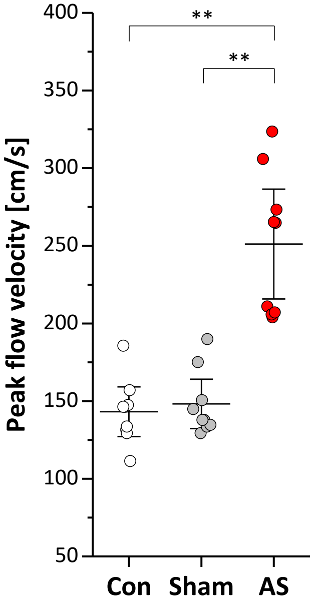

Validation of aortic stenosis by echocardiography

Left: Pulsed wave Doppler in sham-operated (top) and AS (bottom) mice. **Right:** Quantification of peak flow velocity for all groups investigated; n=9 each, $^{**}P<0.01$.
